# The Impact of the COVID-19 Epidemic on Orthodontic Patients in China: An Analysis of Posts on Weibo

**DOI:** 10.3389/fmed.2020.577468

**Published:** 2020-12-08

**Authors:** Feiyang Guo, Bojun Tang, Danchen Qin, Tingting Zhao, Yu-xiong Su, Colman McGrath, Fang Hua, Hong He

**Affiliations:** ^1^Department of Orthodontics, Hubei-MOST KLOS & KLOBM, School & Hospital of Stomatology, Wuhan University, Wuhan, China; ^2^Discipline of Oral and Maxillofacial Surgery, Faculty of Dentistry, The University of Hong Kong, Hong Kong, China; ^3^Applied Oral Sciences & Community Dental Care, Faculty of Dentistry, The University of Hong Kong, Hong Kong, China; ^4^Center for Evidence-Based Stomatology, Hubei-MOST KLOS & KLOBM, School & Hospital of Stomatology, Wuhan University, Wuhan, China; ^5^Division of Dentistry, School of Medical Sciences, Faculty of Biology, Medicine and Health, University of Manchester, Manchester Academic Health Science Centre, Manchester, United Kingdom

**Keywords:** COVID-19, orthodontics, social media, dental care, online health

## Abstract

**Background:** During the COVID-19 pandemic, many dental care services including orthodontic practice were suspended. Orthodontic patients turned to social media platforms to communicate, share experiences, and look for solutions. Our study aimed to investigate the attitudes and perspectives of orthodontic patients during the COVID-19 epidemic in China by analyzing orthodontics-related posts on Sina Weibo (a Chinese counterpart of Twitter).

**Materials and Methods:** Potentially eligible posts on Sina Weibo platform were collected between December 30, 2019, and April 18, 2020. Posts related to both orthodontics and COVID-19 were included and then coded and classified into specific appliances and themes. Geographic and temporal distributions of the included posts were analyzed. In addition, time-lagged cross correlation was performed to explore the association between the number of daily posts and daily new COVID-19 cases/deaths in China. Chi-square tests were employed to compare the differences between fixed appliances and aligners in *problems/difficulties* and *feelings* during the epidemic.

**Results:** Of the 28,911 posts identified, 4,484 were included in the analysis. The most frequently mentioned themes were *appointments* (*n* = 2,621, 58.5%), *negative feelings* (*n* = 2,189, 48.8%), and *problems/difficulties* (*n* = 1,155, 25.8%). A majority of posts were tweeted in regions with high levels of economic development and population density in eastern China and from February to March. The number of daily posts had a significantly positive correlation with daily new COVID-19 cases/deaths in China (*P* < 0.05). Compared with clear aligners, patients with fixed appliances reported more *problems/difficulties* (*P* < 0.001) and *negative feelings* (*P* < 0.001), but fewer *positive feelings* (*P* < 0.001).

**Conclusions:** The analysis of Weibo posts provided a timely understanding of the impact of COVID-19 on orthodontic patients. Delayed appointments were their greatest concern, and negative feelings and untreated orthodontic problems increased during the suspension of dental care services. However, patients with clear aligners reported fewer negative feelings and problems than those with fixed appliances. The findings highlighted the need to consider both treatment- and psychology-related issues of orthodontic patients and how to handle them appropriately during the epidemic.

## Introduction

The coronavirus disease 2019 (COVID-19) caused by severe acute respiratory syndrome coronavirus 2 (SARS-CoV-2) has spread rapidly throughout the world and triggered a global pandemic after being discovered in Wuhan, China, in December 2019 (1). The coronavirus showed high contagiousness and rapid spread, and as of June 9, 2020, the World Health Organization (WHO) reported 7,039,918 confirmed COVID-19 cases and 404,396 deaths (2).

The characteristics of dental practices, such as close face-to-face communication, droplet- and aerosol-generating procedures, and contaminated surfaces, expose dental patients and practitioners to high levels of pathogenic microorganisms and high risks of cross infection (3, 4). In addition, standard precautions are insufficient to protect practitioners and patients from the infection of COVID-19, which brings great challenges to dental services in this period (3). Therefore, most epidemic-attacked regions in China enforced strict regulations to dental health services in late January 2020, which only permitted emergent dental services (4). After 2 months of effective anti-epidemic efforts, China moved into a mitigation stage in April (5), and dental facilities began to gradually resume routine services under strict protective measures.

Scheduled orthodontic appointments and most orthodontic problems were deemed nonemergent; thus, most orthodontic departments and clinics were suspended during the epidemic (6). During the time of orthodontic practice suspension and home quarantine, many orthodontists and patients were at home and communicated by smartphones or online telemedicine services (4, 7). Given the failure to attend regular appointments, orthodontic problems such as loosening of brackets and archwires could not be treated timely in this long period of dental service suspension (6), which had a considerable impact on the lives and treatments of orthodontic patients. Therefore, being aware of patients' mental and physical conditions was necessary for orthodontists to give appropriate suggestions to patients during the epidemic and to prioritize the needs of patients after dental service reopening.

Social media have shown unique advantages in the dissemination of information, and the activities on social media platforms have increased sharply in the special period of transportation restriction and home quarantine (8). As a Chinese counterpart of Twitter, Sina Weibo is a popular social media platform that allows users to share their experiences, opinions, and perspectives, as well as to follow and communicate with others (9). The large scale of users, huge volume of information, and availability of free and immediate access have made it possible for researchers to investigate the public perception of public health events such as MERS-CoV and H7N9 through Sina Weibo (10). In addition, Weibo, regarded as an ideal tool to search large-scale, comprehensive information in a short time, was widely adopted in research related to impact of COVID-19 on public opinions, dental service, and psychology (11–13).

Previous studies have indicated that the COVID-19 epidemic had a negative impact on the public (14, 15). However, to our knowledge, its impact on orthodontic patients has not been explored, and Weibo has not been applied in the orthodontic field before. Therefore, this study aimed to investigate attitudes and perspectives of orthodontic patients during the COVID-19 epidemic, by analyzing the geographic location, time, and content of orthodontic-related posts on Weibo during the COVID-19 epidemic in China.

## Materials and Methods

### Ethics

This study was approved by the Ethics Committee of School & Hospital of Stomatology, Wuhan University (no. 2020-B46). As the study utilized open information on the Internet, personal privacy and clinical data of the research subjects were not involved. Therefore, an exemption for informed consent was granted (11).

### Data Collection

Potentially eligible posts were searched and collected retrospectively by two authors (F.G. and B.T.) from the Sina Weibo platform between December 30, 2019, and April 18, 2020. We used 9 keywords regarding COVID-19 and 10 keywords about orthodontics (Table 1), which were searched in combinations (i.e., one COVID-19 keyword combined with one orthodontic keyword). A Python-based platform Gooseeker was employed to collect all the Weibo posts and extract data from each post, including Weibo number, Weibo username, full-text content, posting date, and location tag (optional when posting). In addition to the location tag, we also extracted geographic location information from full-text posts.

**Table 1:** Keywords for searching the posts (translated from Chinese).

**COVID-19-related keywords**	**Orthodontic-related keywords**
Pneumonia	Orthodontics
Novel coronavirus	Correction
Virus	Orthodontic treatment
Epidemic	Invisalign®^a^
Anti-epidemic	Angelalign®^b^
Epidemic prevention	Braces
Battle against the epidemic	Brackets
COVID-19	Archwires
NCP (novel coronavirus pneumonia)	Oral myofunction
	Sleep disordered breathing

a *Align Technology, Santa Clara, CA, USA*.

b *EA Medical Instruments, Shanghai, China*.

After the collection of posts, duplicate posts were initially removed by Microsoft Excel (2019), and then the remaining posts were screened by two authors (F.G. and D.Q.) to identify eligible posts. As decided *a priori*, only Weibo posts pertaining to both COVID-19 and the users' own orthodontic experience were included. Posts not related to COVID-19 (e.g., other types of pneumonia or other viral infections), irrelevant to orthodontic experience (e.g., prosthodontic treatment or other dental experience), posted by dental professionals, containing advertisements, or having a geographic location outside of China were excluded.

### Coding Procedure

The included posts were read iteratively to familiarize two authors (F.G. and D.Q.) with the contents before coding. Inductive and deductive manual coding approaches were used (16). Initial primary coding categories for type of appliances and post contents were developed based on our study objective and previous research (17–19). Each category had several main themes (Table 2). A pilot coding on a subset of posts (10% randomly selected posts) was then conducted independently and in duplicate by two authors (F.G. and D.Q.) using the predetermined codes. Posts that could not be categorized with the initial codes were given a new code. Any disagreement was resolved via discussion with two experts (F.H. and H.H.). After coding, data were examined to identify potential subcategories within each primary category.

**Table 2:** Main themes and specific appliances for coding with definition and example.

**Category^a^**	**Definition**	**Example**
**Main theme^b^**		
Problems/difficulties	Problems related to orthodontic treatment	“…epidemic… The rubber bands have been used up. And the wire deformed when I just ate the braised chicken.”
Appointments	Able/unable to attend orthodontic appointments	“…I haven't made an appointment of my appliances for 3 months because of the epidemic….”
Negative feelings	Obvious emotional words and/or emoticons expressing negative feelings	“If it wasn't for the epidemic, I would have started orthodontic treatment! I'm mad!!!”
Positive feelings	Obvious emotional words and/or emoticons expressing positive feelings	“…because of this epidemic…Finally I got the new aligners and my treatment will soon come to an end. I can see the dawn of victory!”
Contact with dentists	Able/unable to contact the dentist during the epidemic	“…clinics provided the online follow-up consultation warm-heartedly and professionally, and send new aligners and rubber bands…”
Consultation	Orthodontic consultation with netizens for advice	“I can't see the dentist during the epidemic and found my teeth becoming a little bit irregular. I want to ask what's wrong with my teeth ?”
Orthodontic plans	Plans for orthodontic treatment after the epidemic	“I'm not afraid of tooth extraction, I just want to put on braces after pneumonia”
Others	Other COVID-19- and orthodontic-related posts could not be classified into the above-mentioned themes, e.g., routine records	“Today is my 496th day of wearing Invisalign®. During the epidemic, I persisted in wearing aligners and now I'm wearing the 49th maxillary and 57th mandibular aligner.”
**Specific appliances^c^**		
Fixed appliances	Posts with key words indicating fixed appliances, for example, brackets or archwires	“My brackets are lost! Pain! Please let the epidemic end soon!”
Clear aligners	Posts with key words indicating clear aligners, for example, Invisalign® or Angelalign®	“…This is the 11th week to wear Invisalign®…”
Retainers	Posts with key words indicating retainers	“…My retainer on the table was thrown by others…I can't get another pair during the epidemic”

a *A post could be classified into both main theme and specific appliances*.

b *A post was classified into more than one main theme if containing miscellaneous information*.

c *Only posts that contained key indicating words and could be identified as a specific type of appliances were coded*.

Prior to the formal coding procedure, 60 randomly selected posts were coded by two authors (F.G. and D.Q.) independently and in duplicate to achieve an agreement on the understanding of themes and subthemes. Thereafter, all posts were coded and sorted into primary categories and/or subcategories by the same two authors. If new codes emerged during this procedure, the temporary coding categories would be revised accordingly. All disagreements in classification and naming of themes and subthemes were resolved by discussion with two experts (F.H. and H.H.).

### Data Analysis

Descriptive statistics were performed to summarize the characteristics of the included posts. For geographic distribution analysis, the number of posts with location was compared with the cumulative number of COVID-19 cases in each region of China. Epidemiological data were retrieved from the official website of the Chinese Center for Disease Control and Prevention (CCDC) (5).

Temporal distribution analysis was performed to (1) present the change of number of posts and confirmed new COVID-19 cases/deaths each day in China; (2) display the daily number of posts related to orthodontic problems; (3) compare the daily number of posts of *positive feelings* with that of *negative feelings*; and (4) analyze the number of posts each day related to ability (able or unable) to attend an appointment during the epidemic. Time-lagged cross correlation (TLCC) was used to investigate the association between daily number of posts and daily confirmed new COVID-19 cases/deaths in China.

Chi-square tests were employed to compare the differences between fixed appliances and aligners in *problems* and *positive* and *negative feelings*. IBM SPSS Statistics 23.0 was used for statistical analysis, and a two-tailed *P* < 0.05 was considered significant.

## Results

### Inclusion and Basic Characteristics of Posts

**Figure 1:**
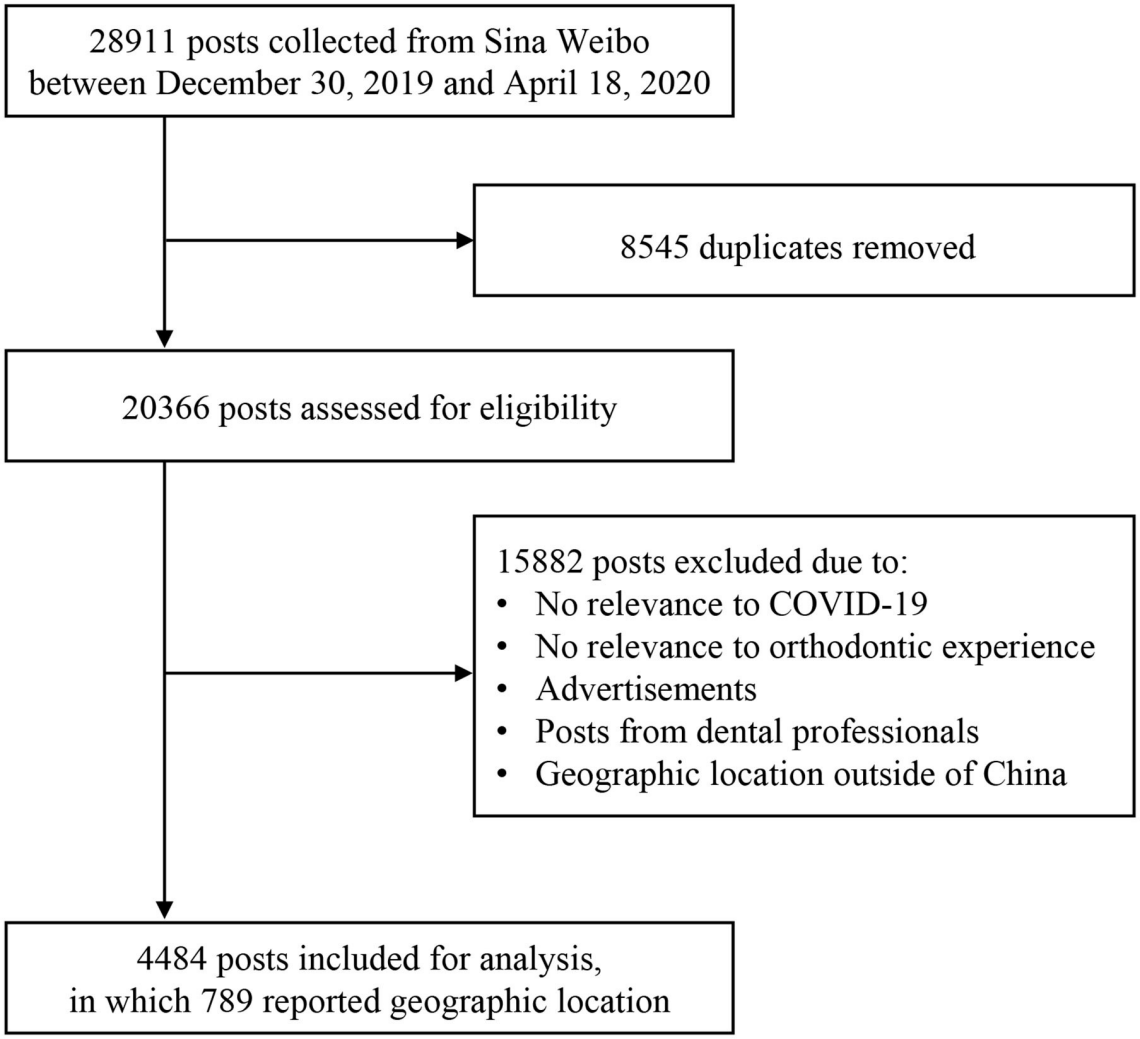
Flow chart of posts selection.

A total of 28,911 Weibo posts were collected, of which 4,484 were finally included in the analysis (Figure 1). Although we identified 305 posts regarding oral myofunction or sleep disordered breathing, none of these posts were concerning orthodontics. Among 4,484 included posts, 1,835 could be identified and coded as a specific type of appliances, in which 786 (42.8%) were related to fixed appliances, 985 (53.7%) clear aligners, and 64 (3.5%) retainers (Table 3).

**Table 3:** Number of posts for specific appliances (*n* = 1,835)^a^.

**Appliances**	**Number**	**Percentage (%)**
Fixed appliances	786	42.8%
Clear aligners	985	53.7%
Retainers	64	3.5%
Total	1,835	100%

a *Among 4,484 included posts, 1,835 could be identified as a specific type of appliances and were coded*.

### Geographic Distribution of Posts

**Figure 2:**
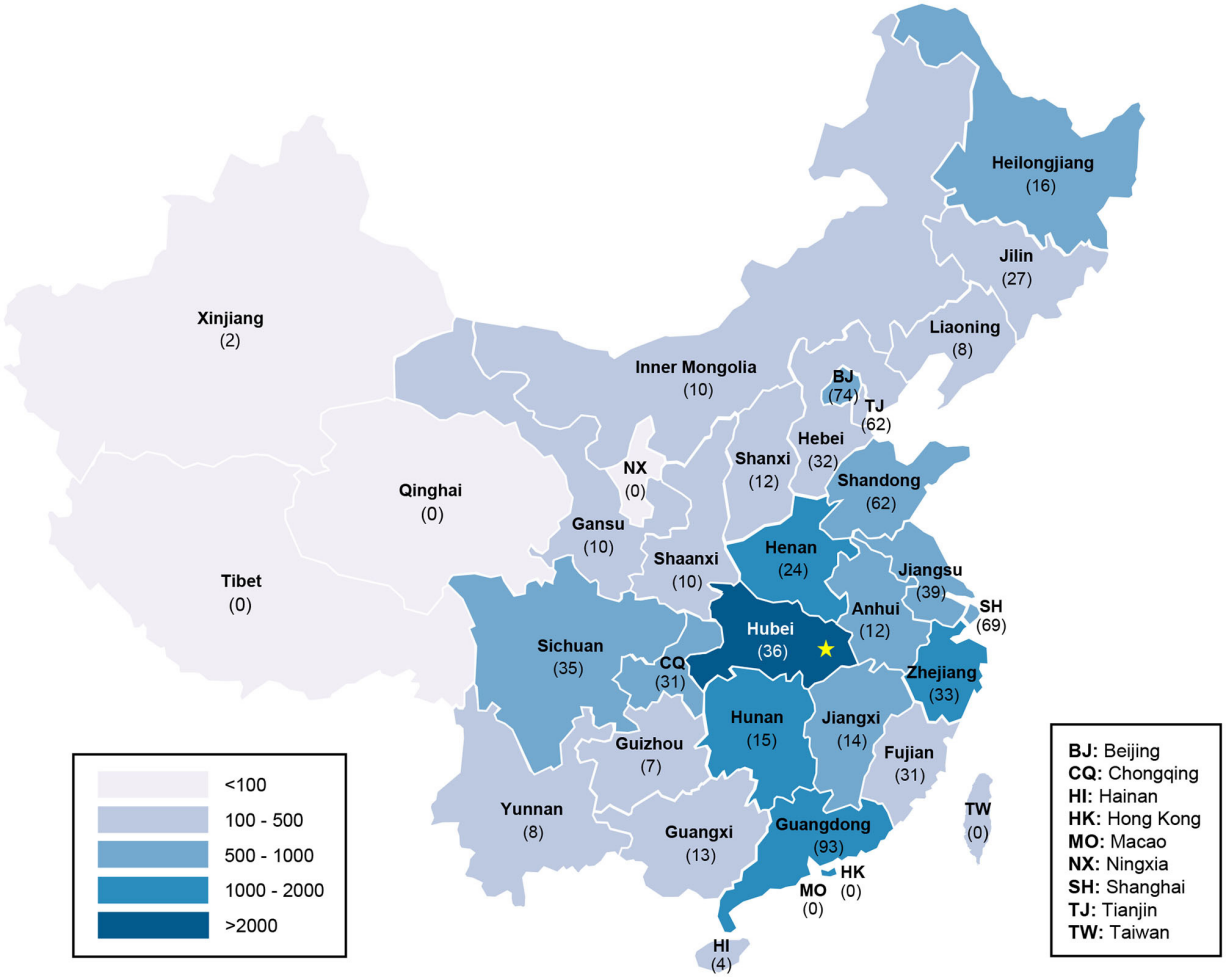
Geographic distribution of Weibo posts and total cases of COVID-19 in China (*n* = 789). The number of posts in each region is presented in parentheses. The gradient of colors displays the cumulative number of confirmed COVID-19 cases (by June 3). The yellow star marks the location of Wuhan.

Among the 4,484 included posts, only 789 (17.6%) reported geographic information. The results of geographic distribution analysis are presented in Figure 2. In western China with <100 COVID-19 cases confirmed, posts were less frequently distributed. More than half of the posts containing location information were posted from eastern China, where four provinces (Guangdong, Beijing, Shanghai, and Shandong) had the most posts (>60). However, <40 posts were from Hubei, the original epicenter and worst-affected province of COVID-19 in China.

### Frequency of Main Themes and Subthemes

**Figure 3:**
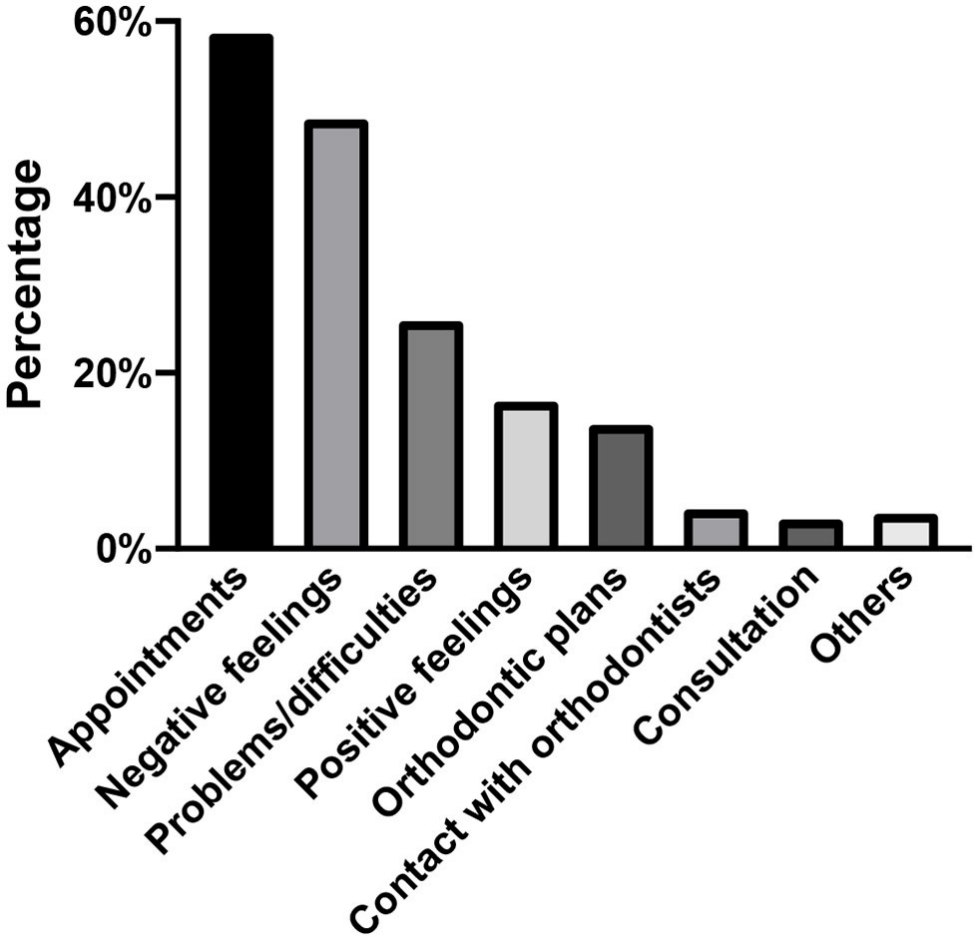
Percentage of posts in each main theme (*n* = 4,484).

Figure 3 illustrates the percentage of posts in each main theme. The most frequently mentioned themes were *appointments* (*n* = 2,621, 58.5%), *negative feelings* (*n* = 2,189, 48.8%), *problems/difficulties* (*n* = 1,155, 25.8%), and *positive feelings* (*n* = 750, 16.7%). *Negative feelings* were reported 3 times more frequently than *positive feelings*. Only a few posts mentioned *contact with dentists* (*n* = 196, 4.4%) and *consultation* (*n* = 146, 3.3%).

The frequency and distribution of subthemes within each main theme are shown in Table 4. Among the 1,155 posts related to *problems/difficulties*, the most commonly listed issue was “loose brackets or archwires” (*n* = 479, 41.5%), followed by “shortage of subsequent aligners” (*n* = 268, 23.2%) and “poking archwires or ligatures” (*n* = 175, 15.2%). In terms of 2,126 Weibo posts regarding *appointments*, three-quarters were regarding “unable to attend an appointment” (*n* = 1,875, 71.5%). However, only a relatively small proportion of posts mentioned being “able to attend an appointment” (*n* = 747, 28.5%). In accordance with the *appointments* and *problems/difficulties*, 2,189 posts expressed feelings in a negative tone, in which most of them were related to treatment delay (*n* = 1,933, 88.3%), followed by “complaint about pain from orthodontic appliances” (*n* = 132, 6.0%).

**Table 4:** Frequency and distribution of subthemes within each main theme (*n* = 4,484).

**Main theme**	**Total**	**Subtheme**	***N* (%)**
Problems/difficulties^a^	1,155	Loose brackets or archwires	479 (41.5%)
		Shortage of subsequent aligners	268 (23.2%)
		Poking archwires or ligatures	175 (15.2%)
		Broken appliances	72 (6.2%)
		Loss of aligners/retainers	55 (4.8%)
		Insufficient rubber bands	34 (2.9%)
		Aligners worn irregularly	32 (2.8%)
		Failed attachments	29 (2.5%)
		Loose temporary anchorage devices	23 (2.0%)
		Unfit aligners	13 (1.1%)
		Others	54 (4.7%)
Appointments	2,621	Unable to attend an appointment	1,875 (71.5%)
		Able to attend an appointment	746 (28.5%)
Negative feelings^a^	2,189	Negative feelings toward treatment delay	1,933 (88.3%)
		Complaint about pain from orthodontic appliances	132 (6.0%)
		Concerns about orthodontic visits during the epidemic	110 (5.0%)
		Fear of the COVID-19 epidemic	23 (1.1%)
		Poor dental care services	20 (0.9%)
		Others	54 (2.5%)
Positive feelings^a^	750	Excellent dental care service	217 (28.9%)
		Pleased to get new aligners during the epidemic	149 (19.9%)
		Looking forward to ending orthodontic treatment	135 (18.0%)
		Pleased to attend an appointment	131 (17.5%)
		Convenience/comfort of clear aligners	115 (15.3%)
		Others	126 (16.8%)
Contact with dentists	196	Able to contact dentists	180 (91.8%)
		Losing contact with dentists	16 (8.2%)

a *A post was classified into more than one subtheme if containing miscellaneous information*.

In 750 posts related to *positive feelings*, one-third were concerning clear aligners, including “pleased to get new aligners during the epidemic” (*n* = 149, 19.9%) and “convenience/comfort of clear aligners” (*n* = 115, 15.3%). “Excellent dental care service,” with key words such as “warm-hearted” and “careful,” was mentioned 10 times more commonly than “poor dental care service” (217 vs. 20). Other reasons for positive feelings were “looking forward to ending orthodontic treatment” (*n* = 135, 18.0%) and “pleased to attend an appointment” (*n* = 131, 17.5%). Moreover, according to 196 posts with respect to *contact with dentists*, most patients could contact their dentists (*n* = 180, 91.8%) and make online consultation or face-to-face appointments with them, whereas a few lost contacts with their dentists (*n* = 16, 8.2%).

### Temporal Distribution of Weibo Posts and Specific Themes

**Figure 4:**
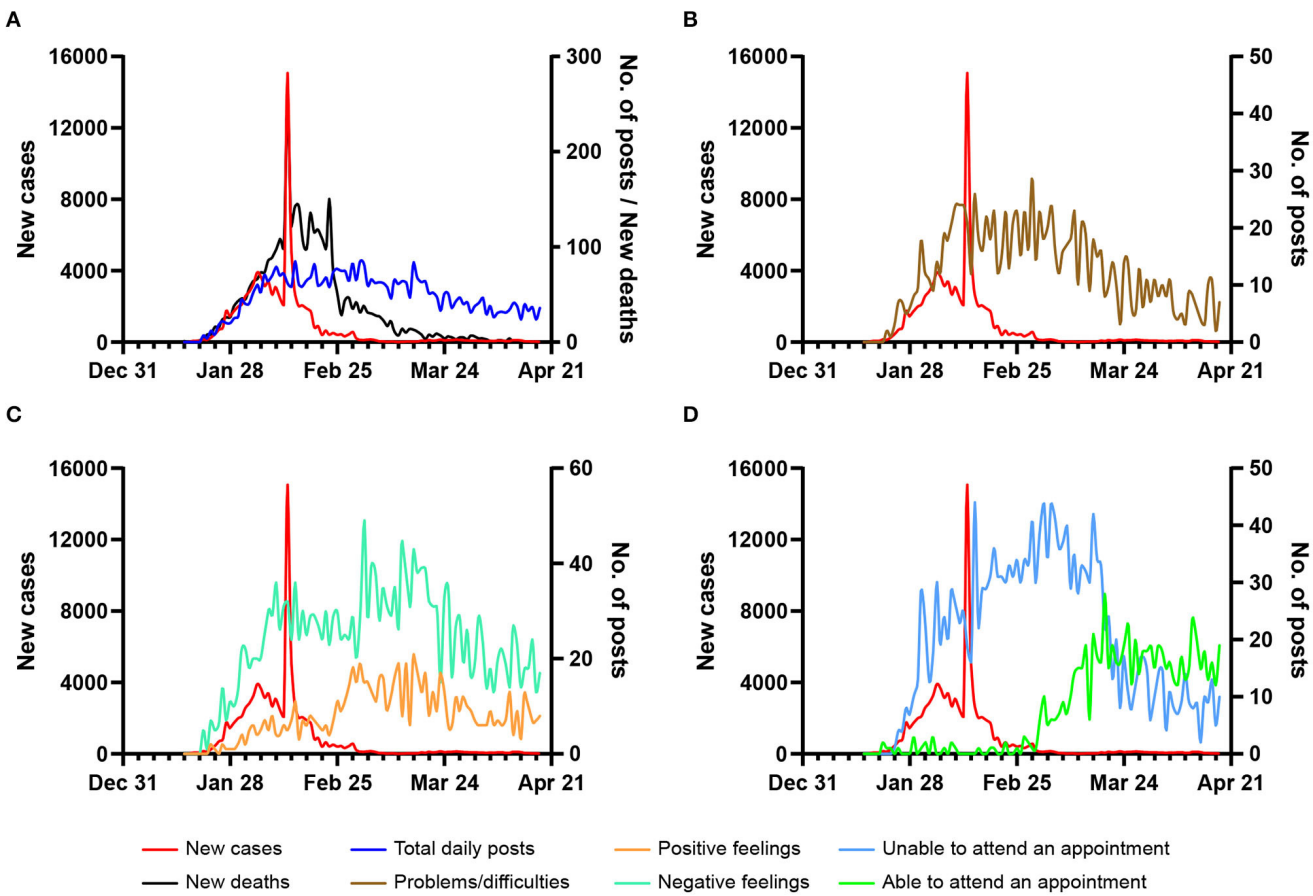
Number of Weibo posts and daily new COVID-19 cases/deaths in China. Total number of posts **(A)**, and number of posts related to problems **(B)**, positive and negative feelings **(C)** and if able to attend an appointment **(D)** are compared with daily new cases/deaths.

The temporal distribution of Weibo posts is presented in Figure 4A. No posts were identified before January 21. Then the number of posts began to increase from late January to early February, which was in accordance with the trend of daily new COVID-19 cases/deaths. The number of posts maintained a steady high level for 2 months, about 60 to 80 posts per day. In late March, the number of posts started to decline gradually.

The trend of posts related to *problems/difficulties* during this period (Figure 4B) was consistent with that of total posts. The number of posts related to orthodontic problems reached a plateau between mid-February and mid-March and then gradually declined. *Positive and negative feelings* showed opposite trends during the epidemic (Figure 4C). Posts related to *negative feelings* increased sharply in late January and early February and decreased gradually in late March, while those regarding *positive feelings* rocketed in late February. Similar trends were seen in *appointments* (Figure 4D), in which posts related to “unable to attend an appointment” increased quickly in late January and decreased in late March and those regarding “able to attend an appointment” started to increase gradually in early March.

As Table 5 shows, the total number of daily posts was significantly correlated with the daily new cases (*r* = 0.323) and deaths (*r* = 0.555) in lag (−2) (*P* < 0.05), which suggested that the increase in the number of posts happened 2 days before that of new cases or deaths. *Negative feelings* also rose 2 days before the increase of deaths (*r* = 0.287, *P* < 0.05) but were not significantly associated with daily new cases. In addition, the number of posts regarding positive feelings showed a significant negative correlation with daily new cases (*r* = −0.331) and deaths (*r* = −0.272) in lag (5), which indicated that *positive feelings* increased 5 days after the decline of daily new cases or deaths.

**Table 5:** Time-lag correlation coefficients between the number of posts and daily new confirmed COVID-19 cases or daily new deaths (*n* = 4,484)^a^.

**Lag**	**−5**	**−4**	**−3**	**−2**	**−1**	**0**	**1**	**2**	**3**	**4**	**5**
**Daily new cases**											
Total number of posts	**0.297**	**0.265**	**0.249**	**0.323^b^**	**0.229**	0.201	0.196	0.149	0.141	0.073	–0.004
Positive feelings	–0.183	–0.174	**−0.238**	–0.141	–0.182	**−0.231**	**−0.241**	**−0.290**	**−0.320**	**−0.275**	**−0.331^b^**
Negative feelings	0.099	0.120	0.074	0.138	0.050	0.079	0.050	–0.019	0.011	–0.052	–0.100
**Daily new deaths**											
Total number of posts	**0.517**	**0.533**	**0.517**	**0.555^b^**	**0.517**	**0.502**	**0.499**	**0.440**	**0.421**	**0.393**	**0.322**
Positive feelings	–0.013	–0.028	–0.097	–0.063	–0.106	–0.148	–0.157	–0.203	**−0.237**	**−0.228**	**−0.272^b^**
Negative feelings	**0.225**	**0.263**	**0.228**	**0.287^b^**	**0.244**	**0.260**	**0.238**	0.175	0.182	0.130	0.086

a *Values in bold are statistically significant (<0.05)*.

b *The largest correlation between the number of posts and daily new confirmed COVID-19 cases or daily new deaths among different time lags*.

### Comparison Between Fixed Appliances and Clear Aligners

As shown in Table 6, 75.4% of the posts regarding fixed appliances reported problems arising during the epidemic and only 35.4% of those regarding clear aligners. The difference in *problems/difficulties* between the two appliances was significant (*P* < 0.001). For positive feelings, 44.9% of aligners expressed *positive feelings*, which were significantly more than that of fixed appliances (8.8%, *P* < 0.001). On the contrary, the difference between two appliances in *negative feelings* was significant (*P* < 0.001), as more fixed appliances (70.9%) were related to *negative feelings* compared with clear aligners (28.9%). Moreover, posts of fixed appliances expressed more *negative feelings* than *positive feelings*, while clear aligners were in the opposite situation.

**Table 6:** Frequency and distribution in different themes between fixed appliances and aligners (*n* = 1,771).

**Themes**	**Fixed appliances (*n* = 786)**	**Aligners (*n* = 985)**	***P***
Problems/difficulties	593 (75.4%)	349 (35.4%)	<0.001
Positive feelings	69 (8.8%)	442 (44.9%)	<0.001
Negative feelings	557 (70.9%)	285 (28.9%)	<0.001

## Discussion

### Orthodontic Services and the COVID-19 Pandemic

The outbreak of COVID-19 has brought about considerable changes to people's daily life. To control and prevent the spread of COVID-19, in late January, Chinese authorities imposed traffic restriction and appealed to the public to take a series of actions, including self-isolation and quarantine, social distancing, and mask-wearing in public places (20). The outbreak of disease and changes in life are reported to have a psychological impact on the public, such as panic, anxiety, and depression (21, 22).

For orthodontic patients, except for the above-mentioned situations, orthodontics-related problems were also their main concerns. Relevant governments in most regions in China set regulations on dental care services during the epidemic, and all elective dental treatments were suspended including orthodontic practice (4, 23). As a result of the regulation of dental practice as well as travel restriction, orthodontic patients could not attend an appointment on a routine basis, leaving problems untreated and with consequences for a prolonged duration of treatment.

About 1 month after the outbreak of COVID-19, as the epidemic was controlled in most provinces, the regulations began to relax and dental care services gradually resumed in these regions. To investigate the attitudes and perspectives of orthodontic patients during the COVID-19 epidemic, we have adopted a new data search platform, social media Weibo. The application of Weibo in orthodontic research can fit well with the COVID-19 epidemic, due to its advantages in studying population-level health behaviors, social opinions, and public responses to health issues in a timely and dynamic way. Therefore, 4,484 included posts in Sina Weibo were collected between December 30, 2019, and April 18, 2020, about 1 month before the outbreak and after the relief of COVID-19, to provide insights into the changes in the influence of COVID-19 on orthodontic patients during the period of the progression and remission of the epidemic.

### Distribution of Weibo Posts

In this study, posts were mainly from eastern regions that generally had a high level of economic development and population density in China. Three province-level regions including Guangdong, Beijing, and Shanghai posted 2 to 3 times more posts than did Hubei, the most virus-hit province. A previous study focusing on the Weibo posts with COVID-19-related oral health information showed a similar geographic distribution (12). Guangdong, Beijing, and Shanghai are the most economically developed and densely populated areas in China, and more people used Weibo to express their opinions during the epidemic (11). In addition, since orthodontic treatment is relatively expensive and not covered under health insurance in China, more people in these regions with better economic condition receive orthodontic treatment; economically developed regions also have more dental hospitals and clinics and therefore have more orthodontic patients (23). As people were more sensitive to epidemic and social events in these areas with a large population, convenient transportation, and high network penetration, negative feelings caused by epidemic were more likely to emerge (11, 14). Thereby, attention should be paid to the psychological management of orthodontic patients from developed regions.

The trend of Weibo posting was associated with the pattern of reports of daily new cases and death cases of COVID-19, which can be divided into three main stages: rising, stable, and falling. This trend was similar to a previous study on H7N9-related posts on Weibo (24). After the official confirmation of human-to-human transmission of COVID-19 on January 20 and the steep rise in the number of newly confirmed cases, the number of daily posts increased substantially from late January to early February. Then the number of posts reached a plateau and lasted until mid-March, about half a month after the initial control of the epidemic. During this period, most dental practices were suspended as well as orthodontic treatment, and orthodontic patients could only consult via online services (4). As the daily new COVID-19 cases decreased to a relatively low level and dental services began to resume in less-affected regions, the number of posts gradually declined.

As aforementioned, time of each fluctuation of the number of posts was related to the date of major events, especially regulations about dental practice. Likewise, during the “hepatitis B vaccine crisis,” the trend of public reactions on Weibo was also influenced by the number of deaths and the suspension of relevant vaccine (25). In addition, we found a moderate positive correlation between the number of posts and negative feelings and that of new cases or deaths 2 days before and a negative association between positive feelings and number of new cases and deaths 5 days after. Our findings suggest that the popularity of Weibo topics related to COVID-19 and orthodontics was associated with the severity of the COVID-19 epidemic (11), and the implementation of public health interventions could have an impact on patients' reaction to their treatment and feelings in outbreaks of diseases (25).

### The Content of Weibo Posts

In our study, the most frequent reasons for tweeting were delayed appointments and expression of negative feelings. Negative feelings were mainly toward treatment delay including worries about missing appointments, annoyance arising from orthodontic problems, and extension of the duration. A recent study has suggested that the COVID-19 epidemic impacted orthodontic appointments and provoked patients' anxiety, and the biggest concern of over half of the patients was also the delay of treatment (26).

Due to the suspension of orthodontic practices during the epidemic, lots of orthodontic problems remained untreated. A previous study showed that the need for orthodontic treatment ranked second in all dental problem-related Weibo posts during the epidemic (12). In our study, a quarter of posts mentioned orthodontic problems, in which loose brackets and archwires were the most common problems. However, some orthodontic procedures that are aerosol generating were still restricted in high-risk regions after the resumption of orthodontic practice. Therefore, to reduce nosocomial infection, orthodontists should give priority to deal with the patients whose treatment would not have aerosol generated after work resumption and avoid appointments with patients whose treatment was restricted (27).

Telemedicine, the approach of online consultation and remote management of patients, developed rapidly and was widely applied during the COVID-19 epidemic (6, 7). However, in this study, a few patients stated that they were unable to contact their dentists, which compromised patients' confidence in treatment (28, 29). Thus, orthodontists should provide patients with correct contact information in case of any emergent situation. In addition, due to the high risk of infection in dental settings, patients may hesitate to attend an appointment after work resumption (26). Therefore, online consultation may still serve as the main communication method at the beginning of work resumption and is also useful in patient triage in this busy time (30).

In summary, our study on the distribution and content of Weibo posts provided an updated understanding of the attitudes and perspectives of orthodontic patients during the COVID-19 epidemic. With accessible resources to acquire timely and large-scale information (13), the social media platform Weibo was recommended to be used by orthodontists to better understand the physical and psychological conditions of orthodontic patients as well as their needs (31). Given the suspension of routine dental practices, more patients with orthodontic problems would seek support and relevant information about treatment through the Internet. However, this online information remains to be doubtful and might be incorrect or unfounded (18). Previous research has indicated that Weibo was more efficient in disseminating information than conventional ways (24). Therefore, orthodontics can use Weibo to spread professional knowledge regarding the management of orthodontic emergencies, such as the use of orthodontic wax for poking archwires for patients, especially for those who lost contact with their orthodontists. Moreover, our findings can provide data based on which patient management during and after the epidemic can be optimized (29). Since adolescents who are a susceptible population of COVID-19 comprise the majority of orthodontic patients and asymptomatic COVID-19 infection may exist after the epidemic, strict infection control measures are necessary for orthodontists (30). Except for the management mentioned above (including restriction on aerosol-generated practices, online consultation, and strict precautions), it is also recommended to schedule patient appointments carefully and reduce the number of patients to avoid crowds gathering/waiting in the consultation area of dental hospitals and clinics.

### Differences Between Fixed Appliances and Clear Aligners

In the present study, clear aligners showed many advantages over fixed appliances during this epidemic; problems with clear aligners were significantly less than those with fixed appliances. Many patients chose fixed appliances due to lower cost and shorter treatment duration (32); however, they experienced high incidence of bracket detachment and poking archwires, which could not be treated timely during the epidemic (27). In contrast, the main concerns of aligners were shortage of aligners and aligner breakage, which were easier to manage during the lockdown period. Patients were required to extend the wearing time of each available aligner and to wear it carefully to avoid breakage, and after work resumption, they could get the remaining aligners on delivery without the need for an appointment (29). As for feelings, there was a significant difference in positive and negative feelings between fixed appliances and aligners, and patients with fixed appliances were more inclined to get anxious. Previous studies also suggested that aligners had less impact on patients' daily life, and patients felt more satisfaction and less discomfort compared to patients with conventional fixed appliances (33, 34). It seems that patients with clear aligners were easier to manage than those with fixed appliances during the COVID-19 epidemic; therefore, more attention should be paid to the management of patients with fixed appliances.

### Limitations

The present study has some limitations. First, the inclusion of Weibo users alone may not reflect the overall situation in the whole population. However, samples from social media should be representative of adolescents, which predominate the orthodontic patients (18). Although there are other popular social media platforms such as WeChat in China, Weibo is the largest one with ample public opinions and accessible information. In addition, Weibo is the most often used platform for discussing breaking news and events, with 550 million monthly active users in the first quarter of 2020 (35). Second, the present study was based only on the Chinese population, which may not reflect populations from other countries. Further research is needed to study the situation in other countries and regions, which may identify cross-cultural differences. Third, data quality issues and privacy issues are frequently mentioned in social media research (36). Since Weibo users can choose to share posts with the public or specific people and only open public data can be searched, some information was inevitably unavailable. However, such selection bias is common in health care-related research using social media (19, 37). So far, there have been no commonly accepted guidelines or consensus about the ethics of user privacy and data use on social media-based studies. In this study, we only collected public information and strictly avoided direct quotations and presentation of any personal information (38).

## Conclusions

The analysis of Weibo posts provided a timely understanding of the impact of COVID-19 on orthodontic patients. Delayed appointments were their greatest concern, and negative feelings and untreated orthodontic problems increased during the suspension of dental care services. However, patients with clear aligners reported fewer negative feelings and problems than those with fixed appliances. The findings highlighted the need to consider both treatment and psychological issues of orthodontic patients and how to handle them appropriately during the epidemic.

## Data Availability Statement

The raw data supporting the conclusions of this article will be made available by the authors, without undue reservation.

## Ethics Statement

The studies involving human participants were reviewed and approved by Ethics Committee of School & Hospital of Stomatology, Wuhan University. Written informed consent for participation was not required for this study in accordance with the national legislation and the institutional requirements.

## Author Contributions

FH: study conception. FG, BT, DQ, TZ, FH, and HH: study design. FG, BT, DQ, FH, and HH: data collection. FG, DQ, and FH: data analysis. TZ, Y-xS, CM, and HH: data interpretation. FG, BT, and DQ: manuscript drafting. TZ, Y-xS, CM, FH, and HH: critical revision of the manuscript. All authors: approval of the final version.

## Conflict of Interest

The authors declare that the research was conducted in the absence of any commercial or financial relationships that could be construed as a potential conflict of interest.
